# Supplementary material for: Development of a computer-aided design software for the quantitative evaluation of aesthetic damage
Source: PLoS One. 2019 Dec 18;14(12):e0226322. doi: 10.1371/journal.pone.0226322 (PMC6919621; doi:10.1371/journal.pone.0226322)
Supplement: S1 Code site — 1. (ZIP) [file pone.0226322.s001.zip › [EESC_jr][Projeto]Relatorio_07_21/vendors/select2/docs/_includes/options/compatibility/initial-selection.html]

## Old initial selections with `initSelection`

Deprecated in Select2 4.0.
This has been replaced by another option and is only available in the
full builds of
Select2.

In the past, Select2 required an option called `initSelection`
that was defined whenever a custom data source was being used, allowing
for the initial selection for the component to be determined. This has
been replaced by the `current` method on the
data adapter.

Key
:   `initSelection`

Value
:   A function taking a `callback`

Adapter
:   `DataAdapter`

Decorator
:   `InitSelection`
